# Hypoxia-mediated CRIP2 activation via NICD1 translocation regulates glycolysis and cell death

**DOI:** 10.1016/j.gendis.2025.101704

**Published:** 2025-06-02

**Authors:** Sunyoung Park, Sang-Ha Baik, Leon F. Palomera, Eunae Kim, Hark Kyun Kim, Jun-Sik Kim, Jong Ho Kim, Chanhee Kim, Thiruma V. Arumugam, Dong-Gyu Jo

**Affiliations:** a School of Pharmacy, Sungkyunkwan University, Suwon 16419, South Korea; b School of Agriculture, Biomedicine and Environment, La Torbe University, Victoria 3086, Australia; c Samsung Advanced Institute for Health Sciences and Technology, Sungkyunkwan University, Seoul 06351, South Korea; d Biomedical Institute for Convergence, Sungkyunkwan University, Suwon 16419, South Korea; e Institute of Quantum Biophysics, Sungkyunkwan University, Suwon 16419, South Korea

Ischemic stroke is a severe neurological condition and a leading cause of disability and death worldwide.^1^ It occurs when blood flow to the brain is critically reduced, depriving cells of oxygen and glucose, which leads to significant cell damage and death. Developing effective therapies for ischemic stroke requires a precise understanding of the downstream mechanisms of Notch intracellular domain 1 (NICD1), which plays a pivotal role in stroke pathology. However, these mechanisms remain only partially understood. Clarifying NICD1 pathways could not only provide promising strategies for stroke therapy but also reveal innovative targets for broader neuroprotective interventions.

We investigated NICD1 nuclear translocation in response to oxygen-glucose deprivation (OGD), a condition mimicking ischemic stroke. Initially, OGD induction was validated by elevated hypoxia inducible factor 1 subunit alpha (HIF-1α) and reduced glucose transporter type 1 (GLUT1) expression, consistent with hypoxic responses. An increase in HES-1, a downstream target of NICD1, further confirmed Notch pathway activation under OGD (Fig. S1A–D).

In HEK293T cells stably expressing NICD1, we observed that NICD1 progressively translocated to the nucleus under OGD conditions and relocated to the cytosol during reperfusion, indicating reversible translocation (Fig. 1A, B; Fig. S1E, F). We also confirmed NICD1 translocation to the nucleus under OGD conditions in the SH-SY5Y cells (Fig. S1G). We compared NICD1 translocation under different conditions: glucose deprivation (GD), hypoxia, or OGD. As a result, NICD1 translocation was more reversible under OGD conditions compared with GD or hypoxia alone, as indicated by its relocation to the cytosol during reperfusion (Fig. 1C–F).

**Figure 1:**
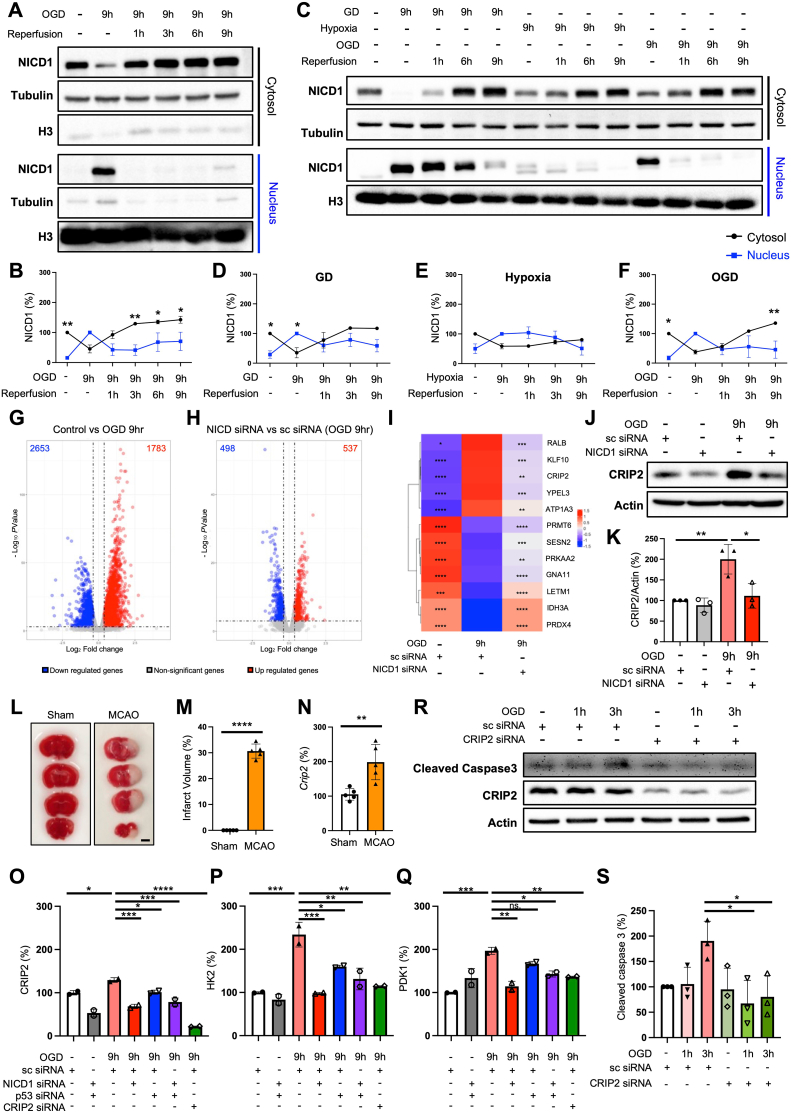
Reversible NICD1 translocation and identification of CRIP2 as a NICD1-regulated target under OGD conditions. **(A, B)** Reversible translocation of NICD1 occurs in a time-dependent manner in HEK293T cells stably transfected with NICD1. NICD1 translocates into the nucleus after 9 h of OGD and subsequently reverts to the cytosol upon re-oxygenation. **(C–F)** The effect of glucose deprivation (GD), hypoxia, and OGD conditions on NICD1 translocation. Two-way ANOVA (Šídák's test) for B, D, E, and F. **(G**–**I)** Identification of CRIP2 as a gene regulated by NICD1 under OGD conditions. (G, H) Volcano plots demonstrate differentially expressed genes (DEGs) between (G) Control versus OGD (9 h), and (H) NICD1 KD versus OGD (9 h). (I) Heatmap of the expression levels of candidate genes. ∗*p* < 0.05, ∗∗*p* < 0.01, ∗∗∗*p* < 0.001, and ∗∗∗∗*p* < 0.0001; analyzed by DESeq2. **(J, K)** Protein quantification of CRIP2 under OGD conditions and NICD1 knockdown (NICD KD) conditions. ∗*p* < 0.05 and ∗∗*p* < 0.01; statistical analysis was performed using one-way ANOVA (Tukey's test). **(L**–**N)** Validation of *Crip2* expression in a mouse model of middle cerebral artery occlusion (MCAO) through quantitative PCR. ∗∗*p* < 0.01 and ∗∗∗∗*p* < 0.0001; statistical analysis was performed using student's *t*-test. Scale bar = 0.25 mm. **(O**–**Q)** CRIP2 knockdown inhibits glycolysis under OGD conditions. The expression levels of (O) CRIP2, (P) HK2, and (Q) PDK1 were confirmed by quantitative PCR. sc siRNA, scrambled siRNA; ∗*p* < 0.05, ∗∗*p* < 0.01, ∗∗∗*p* < 0.001, and ∗∗∗∗*p* < 0.0001; analyzed by ordinary one-way ANOVA (Tukey's multiple comparisons test). **(R, S)** Immunoblots of cleaved capsase-3 protein levels. ∗*p* < 0.05; analyzed by ordinary one-way ANOVA (Tukey's multiple comparisons test). Quantification of western blots (Fig. 1) was performed based on three independent experiments. All western blots were conducted in triplicate, and images for each replicate can be found in Data S1 (Fig. S6–9). Representative images corresponding to Figure 1L for quantifying the infarction volume can be found in Data S1 (Fig. S4A). NICD1, Notch intracellular domain 1; CRIP2, cysteine-rich protein 2; OGD, oxygen-glucose deprivation; HK2, hexokinase 2; PDK1, phosphoinositide-dependent kinase 1.

To investigate the molecular mechanisms underlying NICD1 response under OGD conditions, we conducted transcriptomic analysis to identify genes regulated by NICD1. Transcriptomic comparison between control and OGD conditions revealed 4436 significantly differentially expressed genes (Fig. 1G). Furthermore, we found 1035 differentially expressed genes affected by NICD1 knockdown under OGD conditions (Fig. 1H). Specifically, we identified 5 genes up-regulated under OGD conditions but down-regulated when NICD1 was knocked down (Fig. 1I); and 7 genes down-regulated under OGD conditions but up-regulated when NICD1 was knocked down (Fig. 1I). Among 12 identified genes, validation of mRNA expression levels through quantitative reverse transcription PCR further confirmed that cysteine-rich protein 2 (CRIP2) was particularly sensitive to NICD1 regulation under OGD conditions (Fig. S2A–L).

Additionally, CRIP2 protein levels were reduced following NICD1 knockdown in both control and OGD conditions (Fig. 1J, K) in HEK293T cells stably expressing NICD1. These findings suggest that NICD1 positively regulates CRIP2 expression under ischemic-like conditions, highlighting CRIP2 as a key NICD1 target relevant to stroke pathology. Moreover, NICD1 overexpression in SH-SY5Y cells led to an increase in CRIP2 expression, further supporting the role of NICD1 in regulating CRIP2 levels (Fig. S3). To determine whether *Crip2* expression was altered in an *in vivo* stroke model, we utilized a transient middle cerebral artery occlusion (tMCAO) mouse model. Following surgery, infarct volume increased, confirming successful stroke induction (Fig. 1L, M). Notably, *Crip2* expression was up-regulated in the infarct region (Fig. 1N). These findings collectively suggest that NICD1-mediated regulation of CRIP2 plays a crucial role in ischemic conditions, potentially contributing to stroke pathology through its effects on cellular responses in both *in vitro* and *in vivo* models.

Our findings confirm that NICD1 regulates CRIP2 expression. Additionally, CRIP2 expression is known to be regulated by p53.^2^ To assess whether NICD1 or p53 played a more prominent role in regulating CRIP2 under OGD conditions, we conducted selective knockdowns. Specifically, we knocked down NICD1, p53, or both simultaneously and measured CRIP2 expression levels under each condition. The results showed that CRIP2 expression was down-regulated under all knockdown conditions; however, the most substantial decrease in CRIP2 expression was observed when only NICD1 was knocked down. This suggests that while both NICD1 and p53 influence CRIP2 expression, NICD1 may have a more dominant regulatory role under OGD conditions (Fig. 1O).

We next examined the role of CRIP2 in metabolic adaptation under OGD conditions. Key enzymes in the glycolysis pathway, such as hexokinase 2 (HK2) and phosphoinositide-dependent kinase 1 (PDK1), are known to be up-regulated in response to hypoxia.^3^^,^^4^ To explore the relationship between CRIP2 and glycolysis, we conducted quantitative mRNA analysis of HK2 and PDK1. Both enzymes exhibited elevated expression under OGD conditions, indicating enhanced glycolytic activity (Fig. 1P, Q). Down-regulation of CRIP2 through NICD1 knockdown or p53 knockdown resulted in reduced mRNA levels of HK2 and PDK1, mirroring the effects observed with direct CRIP2 knockdown (Fig. 1P, Q). Based on this, we found that CRIP2 expression levels exhibit a positive correlation with HK2 and PDK1 expression. Furthermore, we confirmed a significant increase in *Hk2* expression in tMCAO animal models (Fig. S4B). These results indicate that CRIP2 plays an essential role in regulating glycolysis under OGD conditions (Fig. S6).

In addition, CRIP2 appears to influence cell death under ischemic conditions. Previous studies have demonstrated that CRIP2 up-regulates cleaved caspase-3,^5^ a marker of apoptosis. Consistent with previous reports, we found that cleaved caspase-3 levels increased in a time-dependent manner under OGD conditions but decreased when CRIP2 was knocked down (Fig. 1R, S). These observations suggest that CRIP2 promotes cell death under ischemic conditions, likely through its regulation of caspase-3.

To further understand the role of CRIP2 in the cell death pathway, we observed that CRIP2 overexpression increased LC3B levels (Fig. S5A) and enhanced autophagic flux, as measured by RFP-GFP tandem fluorescent LC3 (tfLC3) assay (Fig. S5B, C). Furthermore, autophagy can promote apoptosis and induce cell death in cells with defective apoptotic mechanisms, and excessive autophagy has been shown to trigger cell death. Many autophagy-related components are essential for apoptotic factors to mediate cell death, emphasizing the complex crosstalk between these pathways. Our findings of increased autophagic flux upon CRIP2 overexpression provide additional mechanistic insight into CRIP2's role in OGD-induced cell death.

Our findings underscore the critical role of NICD1 in ischemic stroke pathology through its regulation of CRIP2. This study reveals that CRIP2, a downstream target of NICD1, drives glycolytic adaptation and influences apoptosis under OGD (Fig. S6). While our study establishes NICD1 and CRIP2 as key players in ischemic pathology, further work is needed to clarify their exact molecular roles. Specifically, the mechanisms by which NICD1 regulates CRIP2 and how CRIP2 modulates glycolytic and apoptotic pathways remain unclear.

Nevertheless, further research is necessary to elucidate the molecular mechanisms underlying CRIP2 regulation and its impact on ischemic stroke. Although we propose that CRIP2 regulates glycolysis and cell death, this hypothesis merely suggests a possibility rather than confirming the detailed mechanisms involved. Future studies should focus on elucidating these mechanisms and validating our findings, with a particular emphasis on *in vivo* models. Moreover, it is imperative to ascertain the potential adverse effects and constraints of targeting CRIP2 for the treatment of ischemic stroke.

## CRediT authorship contribution statement

**Sunyoung Park:** Writing – review & editing, Writing – original draft, Validation, Investigation, Formal analysis, Data curation, Conceptualization. **Sang-Ha Baik:** Writing – review & editing, Writing – original draft, Visualization, Validation, Investigation, Formal analysis, Data curation, Conceptualization. **Leon F. Palomera:** Writing – review & editing, Validation, Investigation. **Eunae Kim:** Writing – review & editing, Investigation. **Hark Kyun Kim:** Writing – review & editing, Investigation. **Jun-Sik Kim:** Writing – review & editing, Investigation. **Jong Ho Kim:** Writing – review & editing, Investigation. **Chanhee Kim:** Writing – review & editing, Investigation. **Thiruma V. Arumugam:** Writing – review & editing, Writing – original draft, Project administration, Funding acquisition, Data curation, Conceptualization. **Dong-Gyu Jo:** Writing – review & editing, Writing – original draft, Supervision, Project administration, Funding acquisition, Data curation, Conceptualization.

## Ethics declaration

Mouse experiments were performed in accordance with the Basel Declaration and approved by the Institutional Animal Care and Use Committee (IACUC) of the institution (SKKUIACUC2021-10-07-1).

## Data availability

The data that support the findings of this study are available upon request from the corresponding author.

## Funding

This study was supported by the Basic Science Research Program through the 10.13039/501100003725National Research Foundation of Korea (No. RS-2024-00399237, RS-2024-00345742).

## Conflict of interests

The authors declared no competing interests.
