# Supplementary material for: Hypoxia-mediated CRIP2 activation via NICD1 translocation regulates glycolysis and cell death
Source: Genes Dis. 2025 Jun 2;13(2):101704. doi: 10.1016/j.gendis.2025.101704 (PMC12805395; doi:10.1016/j.gendis.2025.101704)
Supplement: Multimedia component 1 [file mmc1.docx]

**Supplementary file 1**

**Materials & methods**

**Cell culture and generation of stably transfected cell lines**

HEK293T cells and SH-SY5Y cells were purchased from ATCC and maintained in Dulbecco's Modified Eagle Medium (DMEM; Hyclone, Logan, Utah, USA), supplemented with 10% fetal bovine serum (FBS; Gibco, Carlsbad, CA, USA) and 1% penicillin/streptomycin (PS; Capricorn Scientific, Germany). HEK293T cells stably expressing Flag-Streptavidin binding peptide-tagged human NICD1 (HEK293T-hNICD1-Flag-SBP Tag) and SH-SY5Y cells stably expressing RFP-GFP tandem fluorescent LC3 (tfLC3) were generated via transfection. All cells were cultured in a humidified incubator at 37°C with 5% CO_2_.

**Oxygen-glucose deprivation (OGD)**

OGD was performed as previously described ^1^. For glucose deprivation, cells were incubated in Locke's buffer for 1, 3, 6, and 9 hours. For hypoxia induction, cells were placed in an oxygen-free chamber for the same durations, following the infusion of N_2_ gas (95% N_2_ + 5% CO_2_) for 5 minutes at a flow rate of 20 L/min. For OGD, cells were treated with Locke's solution and then placed in the oxygen-free chamber for 1, 3, 6, and 9 hours.

**Western blotting**

Protein samples were processed as previously described ^2^. Total protein was extracted from cells using a tissue protein extraction buffer mixture (Thermo Fisher Scientific, Waltham, MA, USA), supplemented with protease inhibitor cocktail and phosphatase inhibitor cocktails (Thermo Fisher Scientific). Protein content was quantified using the Pierce BCA assay (Thermo Fisher Scientific). Equal amounts of proteins were mixed with NuPAGE LDS sample buffer (Thermo Fisher Scientific) containing 5% β-mercaptoethanol. The samples were loaded onto SDS-PAGE gels (Bio-Rad Laboratories, Hercules, CA, USA) and transferred onto 0.45 μm polyvinylidene difluoride membranes (PVDF; Merck Millipore Ltd., Burlington, MA, USA). Membranes were incubated overnight at 4°C with primary antibodies, including anti-HIF-1α (Merck Millipore), anti-Glut1 (Cell Signaling Technology, Danvers, MA, USA), anti-Hes-1(Santa Cruz Biotechnology, Santa Cruz, CA, USA), anti-Flag (Sigma-Aldrich Co., St. Louis, MO, USA), anti-Notch1 (Biolegend, San Diego, CA, USA), anti-HIF1α (Novus biological, Denver, CO, USA), anti-p53 (Santa Cruz Biotechnology), anti-Tubulin (Cell Signaling Technology), anti-H3 (Cell Signaling Technology), anti-beta-actin (Sigma-Aldrich), anti-cleaved caspase-3 (Cell Signaling Technology), anti-CRIP2 (Abcam, Cambridge, UK), anti-G9a (Cell Signaling Technology), anti-Pin1 (Abcam), and anti-LC3 (Cell Signaling Technology). Membranes were then incubated with peroxidase-conjugated anti-rabbit or anti-mouse secondary antibodies (Merck Millipore) for 1 hour at room temperature. Immunoreactive protein bands were visualized using the Dyne ECL STAR Western Blot Detection Kit (Thermo Fisher Scientific).

**Subcellular Fraction**

All samples were maintained on ice throughout the procedure, and all centrifugations were carried out at 4°C. Cells were initially washed twice with cold 1x phosphate-buffered saline (PBS) and then collected in a 1.5-ml tube in PBS, followed by centrifugation for 3 minutes at 2,000 g. Lysis Buffer 1 was added to the pellet, rotated for 5 minutes at 4°C, and centrifuged for 5 minutes at 2,000 g. The resulting supernatant was collected and labeled as 'Cytosol'. The pellet was washed twice with Lysis Buffer 2, centrifuged again for 5 minutes at 2,000 g, resuspended in Lysis Buffer 3, treated with Benzonase nuclease overnight at 4°C, and collected as the supernatant after a final centrifugation for 10 minutes at 2,000 g. This sample, labeled as 'Nuclear'.

**Overexpression and knock down**

For overexpression experiments, cells were transfected with 1 μg of plasmid DNA using PEI (polyethylenimine) transfection reagent and incubated for 24 hours. For knockdown experiments, cells were transfected with 50 nM of siRNA targeting NICD1 (Santa Cruz, CA, USA, sc-36095), p53 (Santa Cruz sc-29435), or CRIP2 (Santa Cruz sc-106947) for 48 hours, following the manufacturer’s protocol.

**RNA extraction and RT-qPCR**

Total RNA was extracted using RNAiso Plus solution (TaKaRa, Shiga, Japan) according to the manufacturer's instructions [16]. RNA aliquots (1000 ng) were used for complementary DNA (cDNA) synthesis (TaKaRa). Real-time reverse transcription PCR (RT-PCR) was conducted using a CFX Connect instrument (Bio-Rad, USA). PCR amplification was carried out with SYBR Premix Ex Taq II (TaKaRa) and PrimePCR™ SYBR® Green (Bio-Rad) under the following conditions: 95°C for 30 seconds, followed by 40 cycles at 95°C for 5 seconds and 60°C for 30 seconds. ΔCt values were calculated to determine differences between experimental and reference genes. Primers for *RALB, KLF10, YPEL3, ATP1A3, PRMT6, SESN2, PRKAA2, GNA11, LETM1, IDH3A,* and *PRDX4* were purchased (PrimePCR™ SYBR® Green Assay, Bio-Rad). Specific primers used included: for human *CRIP2*, forward TGACGTCTCTGGGCAAGGATTG and reverse CCGAAGAGGATTTCCATAGCAGG; for human *NICD1*, forward GGGGCCCTGAATTTCTACTGT and reverse CCCGAGGGGTTGTATTGGT; for human *p53*, forward CCTCTCCCCAGCCAAAAGAG and reverse TCTCGGAACATCTCGAAGCG; for human *HK2*, forward TCTACATAAGACCGTCGCGGC and reverse TAAGGCCATCGTCTCCACCAT; for human *PDK1*, forward ACGGATCAGAACCCGACACA and reverse ACATTCTGGTGTGCACAGG; for human *ACTB* forward CCTGGCACCCAGCACAAT and reverse GGGCCGGACTCGTCATAC; for mouse *Crip2*, forward ATCTACGACAAGGACCCGGA and reverse GAGGCAGGACTAGGCAACAG; for mouse *Hk2*, forward TCTACATAAGACCGTGCGGC and reverse TAAGCCACTGCTGTCACCAT; for mouse *Pdk1*, forward ACGGATCAGAAACCGACACA and reverse ACATTCTGGCTGGTGACAGG; and mouse *Actb* forward CTAAGGCCAACCGTGAAAAG and reverse ACCAGAGGCATACAGGGACA.

**RNA-seq and data processing**

Sequencing analysis was performed as previously described ^3^. The Homo sapiens reference genome (hg19) was indexed, and paired-end quality-checked reads were mapped to it using the STAR aligner^4^. Reads mapping to each gene were quantified using RSEM ^5^. Differential gene expression was analyzed using the DESeq2 R package (v1.42.1) ^6^. Genes with an absolute fold change >1.3 and an adjusted p-value <0.05 were identified as differentially expressed. Heatmaps were created using the ComplexHeatmap R package (v2.18.0) with DESeq2 normalized values. Volcano plots were generated using the ggplot2 R package (v3.5.0). KEGG (Kyoto Encyclopedia of Genes and Genomes) provided pathway information, and DAVID (http://david.ncifcrf.gov) facilitates gene functional analysis.

**Middle cerebral artery occlusion (MCAO)**

Ten-week-old C57BL/6 wild-type mice underwent MCAO as described previously ^7,8^. Transient MCAO was performed using a silicone-coated 6-0 monofilament (Doccol Corporation, Sharon, MA, USA) for 1 hour. Mice were anesthetized with isoflurane (2% v/v in oxygen) during the procedure and temperature was maintained at 37 ºC. Next, analgesia was provided (acetaminophen, 200 mg/kg), and were monitored until awake. Twenty-four hours after reperfusion, mice neurological score was assessed and graded on a 0-5 scale, and those with a score <1 were excluded. Next, mice were euthanized by cervical dislocation. Brains were carefully removed, placed in ice-cold PBS, and cut into 2-mm coronal sections for immediate analysis of infarct volume and processing for RNA extraction as previously reported ^9^.

**Quantification of cerebral infarction**

Coronal brain slices were stained with 2% 2,3,5-Triphenyltetrazolium chloride (TTC; Sigma-Aldrich Chemical Co., St. Louis, MO, USA) at 37°C for 15 min, photographed, and analyzed using NIH FIJI image software. Infarct volume was calculated as (%) = (∑i(Ci - Ni) / 2∑iCi) × 100 (contralateral hemisphere - undamaged ipsilateral tissue) to correct for swelling. Infarct volume was determined by integrating areas across all slices.

**Data analysis**

All statistical analyses were conducted using Prism9 software (GraphPad Software). Two-way ANOVA with Šídák’s multiple comparisons test was used for analyzing the interacting effects between two independent variables on the dependent variable, followed by post-hoc tests to identify significant differences between specific groups when necessary. One-way ANOVA with Tukey's multiple comparisons test was used for comparing more than three groups, while unpaired Student's t-tests assessed significance between two groups. Data are presented as the mean ± standard deviation (s.d.). Significance was determined with p-values <0.05 (*P < 0.05; **P < 0.01; ***P < 0.001).

**Supplementary Figures**


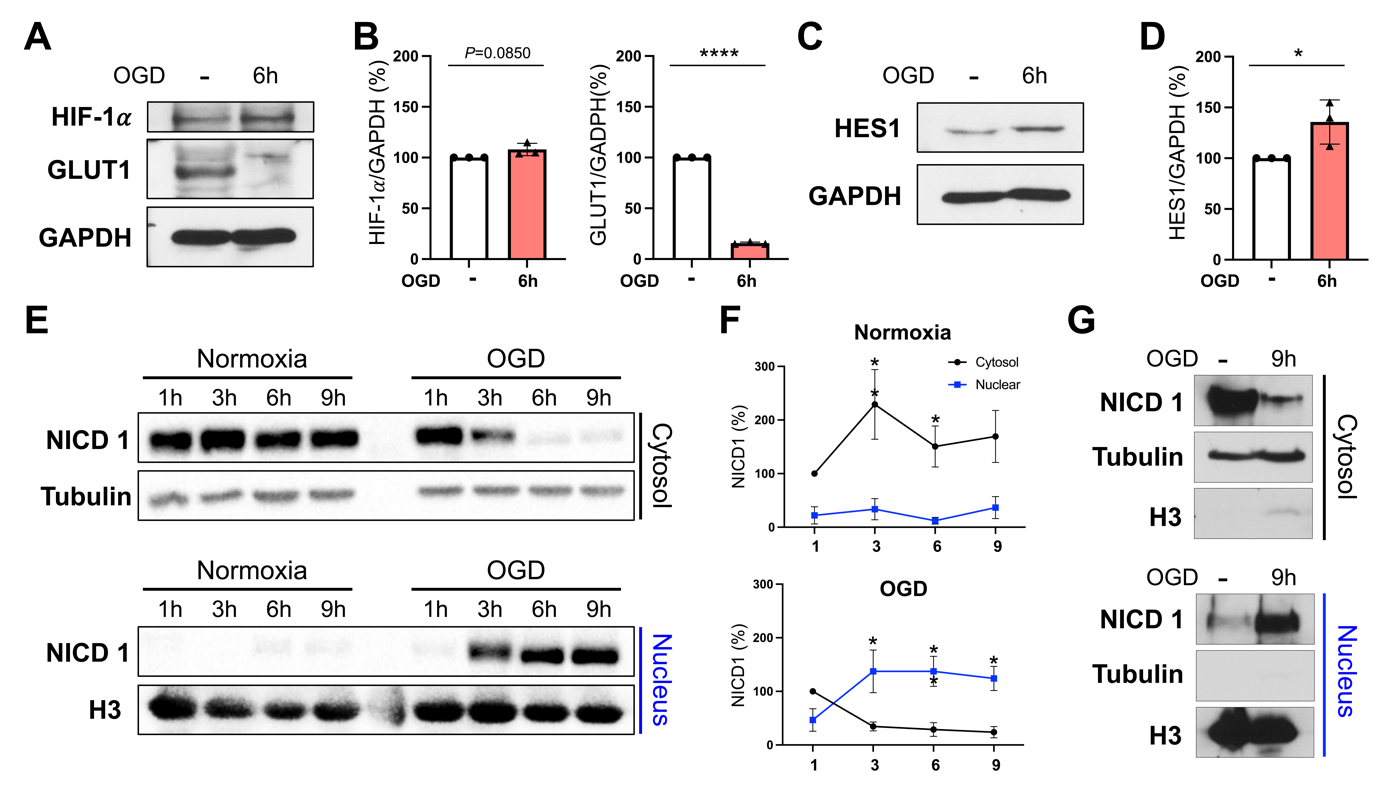


Figure S1. Validation of OGD conditions and translocation of NICD1.

(A–F) Protein quantification and immunoblot analysis under OGD conditions in HEK293T cells. (A, B) Protein quantification of HIF-1𝛼 and GLUT1 under OGD conditions. (C, D) Protein quantification of HES-1 under OGD conditions. (E, F) Immunoblots display NICD1 protein levels at different time points during OGD. (G) Immunoblots showing NICD1 protein levels under OGD conditions in SH-SY5Y cells. *P < 0.05, **P < 0.01, ****P < 0.0001; statistical analysis was performed using student’s t-test for B and D, and two-way ANOVA (Šídák’s test) for F. All Western blots were conducted in triplicate, and images for each replicate can be found in Supplementary Data1 (Fig. S12-S13).


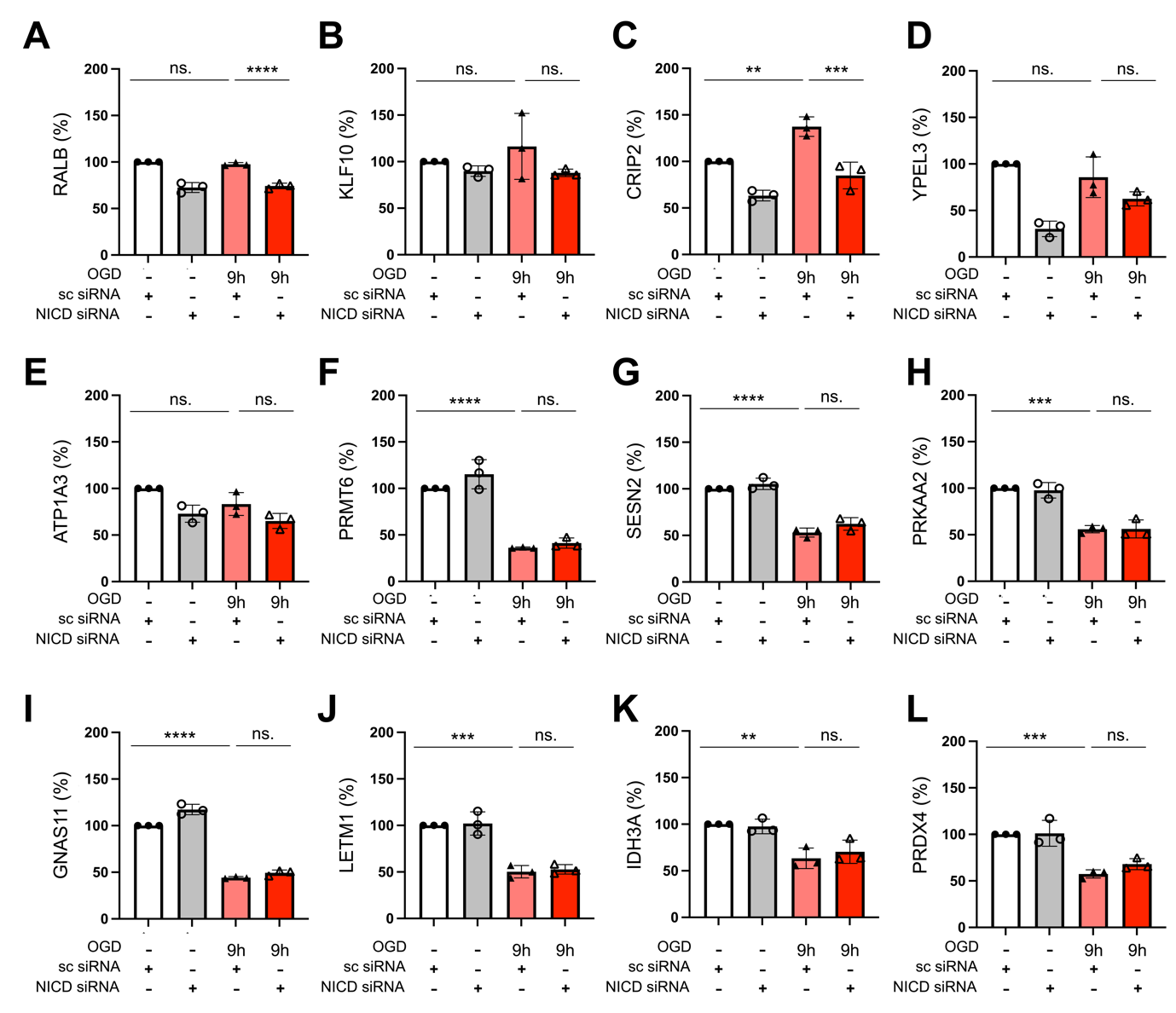


Figure S2. qPCR validation of expression levels for candidate genes under OGD conditions.

The expression level of candidate genes; (A) *RALB*, (B) *KLF10* (C) *CRIP2*, (D)*YPEL3*, (E) *ATP1A3*, (F) *PRMT6*, (G) *SESN2*, (H) *PRKAA2*, (I) *GNA11*, (J) *LETM1*, (K) *IDH3A*, and (L) *PRDX4* confirmed by qPCR. **P < 0.01, ***P<0.001, ****P < 0.0001; statistical analysis was performed and analyzed by Ordinary one-way ANOVA (Tukey’s multiple comparisons test).


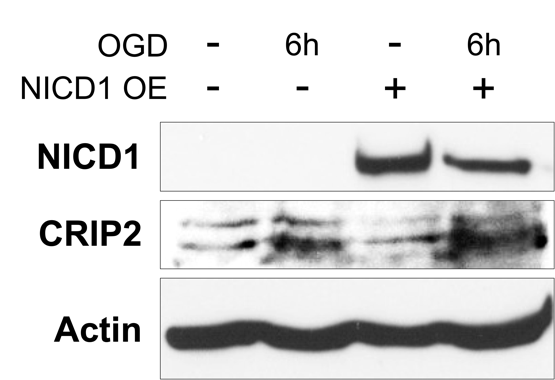


Figure S3. Validation of CRIP2 expression in SH-SY5Y cells under OGD conditions and NICD1 overexpression. NICD1 OE (NICD1 overexpression).


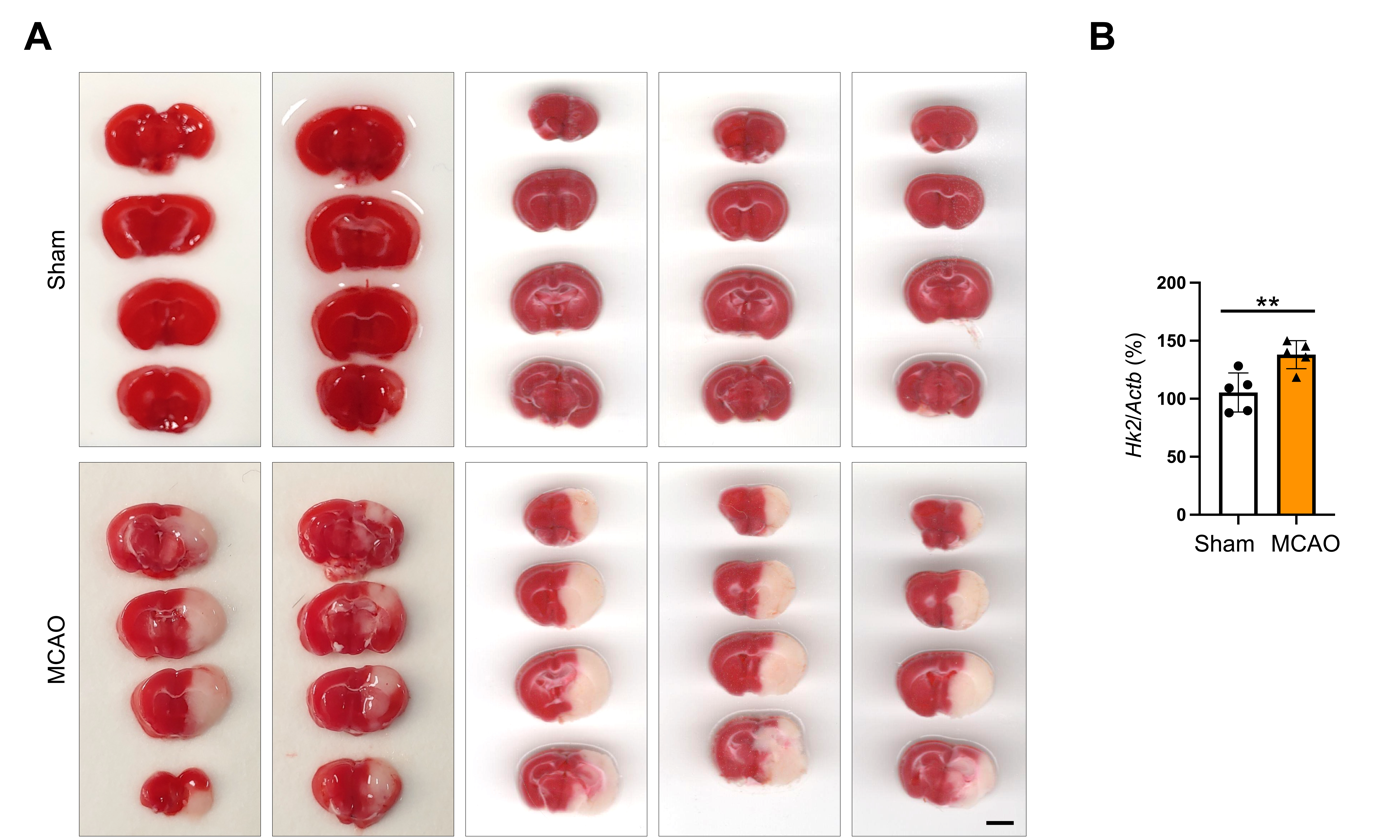


Figure S4. Infarct volume and *Hk2* expression in the MCAO *in vivo* Model. (A) Representative TTC staining images, (B) *Hk2* expression levels in the MCAO *in vivo* model. Scale bar = 0.25mm. **P < 0.01; statistical analysis was performed using student’s t-test.


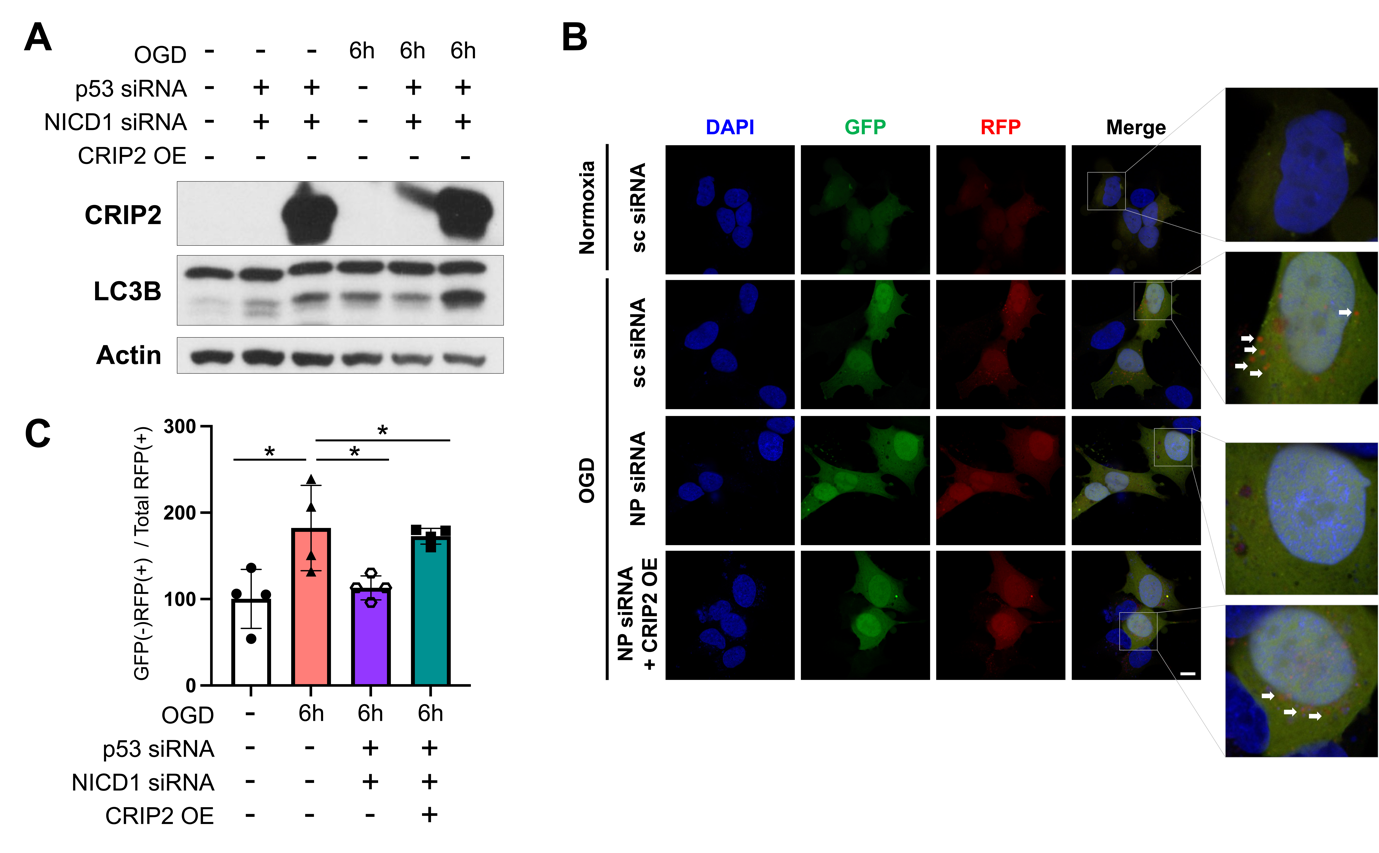


Figure S5. CRIP2 regulates autophagic flux under OGD conditions. (A) Immunoblots showing LC3B-II and CRIP2 protein levels under OGD conditions in HEK293T cells. (B-C) Quantification of autophagic flux in SH-SY5Y cells stably expressing LC3-RFP-GFP. Scale bar = 10 µm. *P < 0.05; statistical analysis was performed analyzed by Ordinary one-way ANOVA (Tukey’s multiple comparisons test).


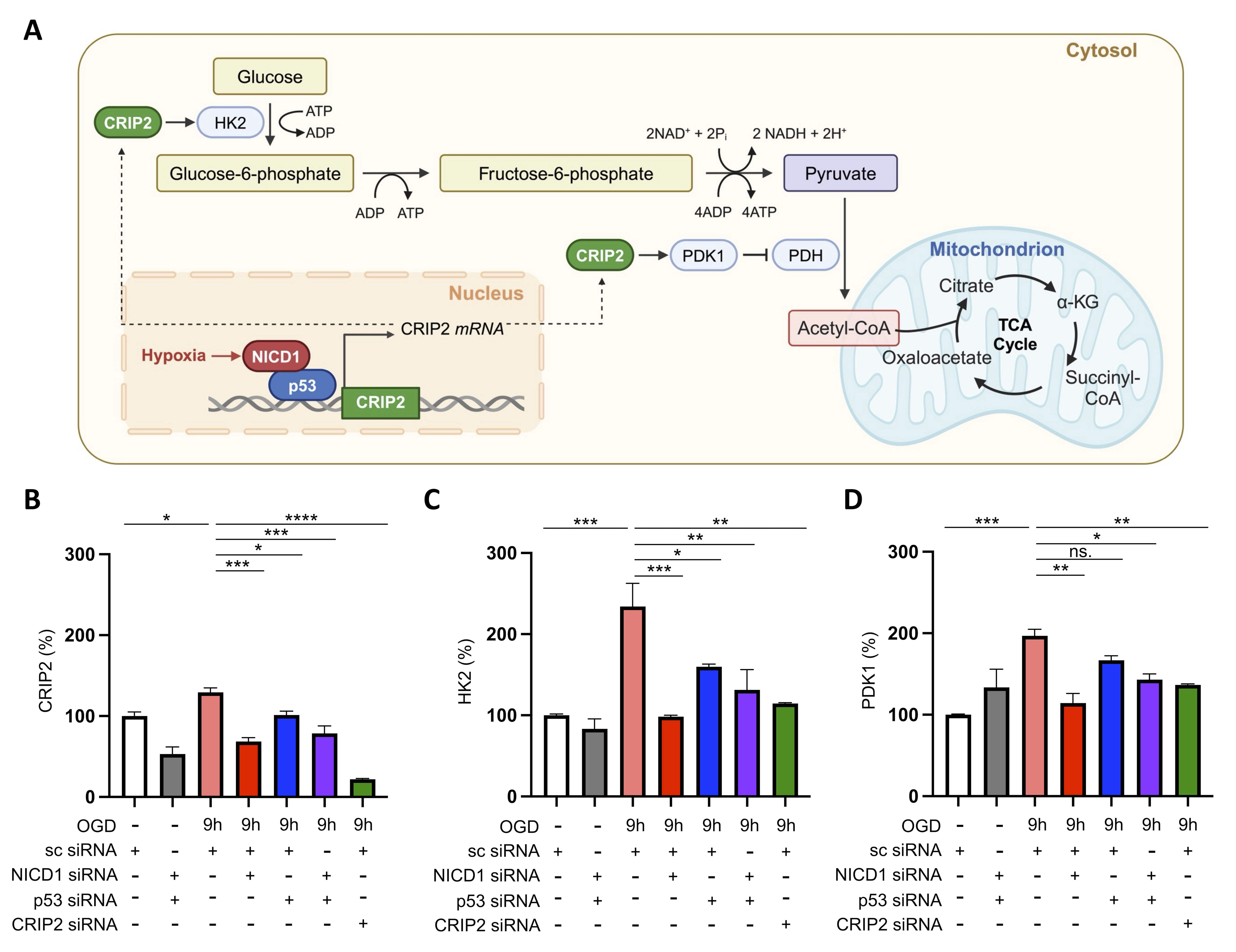


Figure S6. Schematic representation of the glycolysis pathway, highlighting the role of CRIP2 and key enzymes HK2 and PDK1.


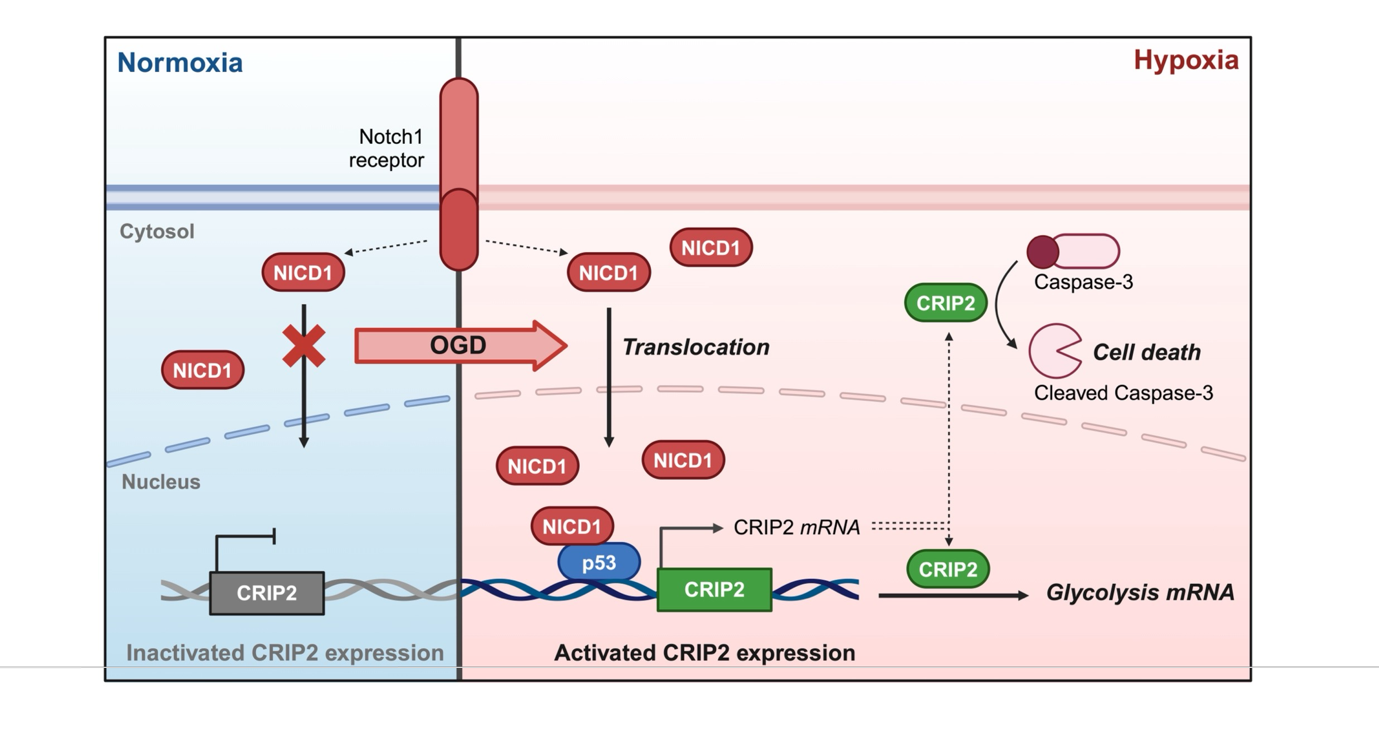


Figure S7. CRIP2 plays a crucial role in cellular pathway under OGD conditions.

OGD conditions trigger reversible NICD1 translocation, activating CRIP2, which in turn drives glycolysis and cell death.

Figure S8. Reversible translocation of NICD1 occurs time-dependently in HEK293T cells stably transfected with NICD1.

Western blot quantification (Fig1. B) was based on three independent experiments, with (A), (B), and (C) representing each replicate.

Figure S9. The effect of glucose deprivation (GD), hypoxia, and OGD conditions on NICD1 translocation. Western blot quantification (Fig1. D-F) was based on three independent experiments, with (A), (B), and (C) representing each replicate.

Figure S10. Protein quantification of CRIP2 under OGD conditions and NICD1 Knockdown (NICD KD) conditions. Western blot quantification (Fig1. J) was based on three independent experiments.

Figure S11. Immunoblots displaying cleaved capsase3 protein levels. Western blot quantification (Fig1. R) was based on three independent experiments, with (A), (B), and (C) representing each replicate.

Figure S12. Protein quantification of HIF-1𝛼, GLUT1, and Hes-1 under OGD conditions. Western blot quantification (Fig. S1B, D) was based on three independent experiments, with (A), (B), and (C) representing each replicate.


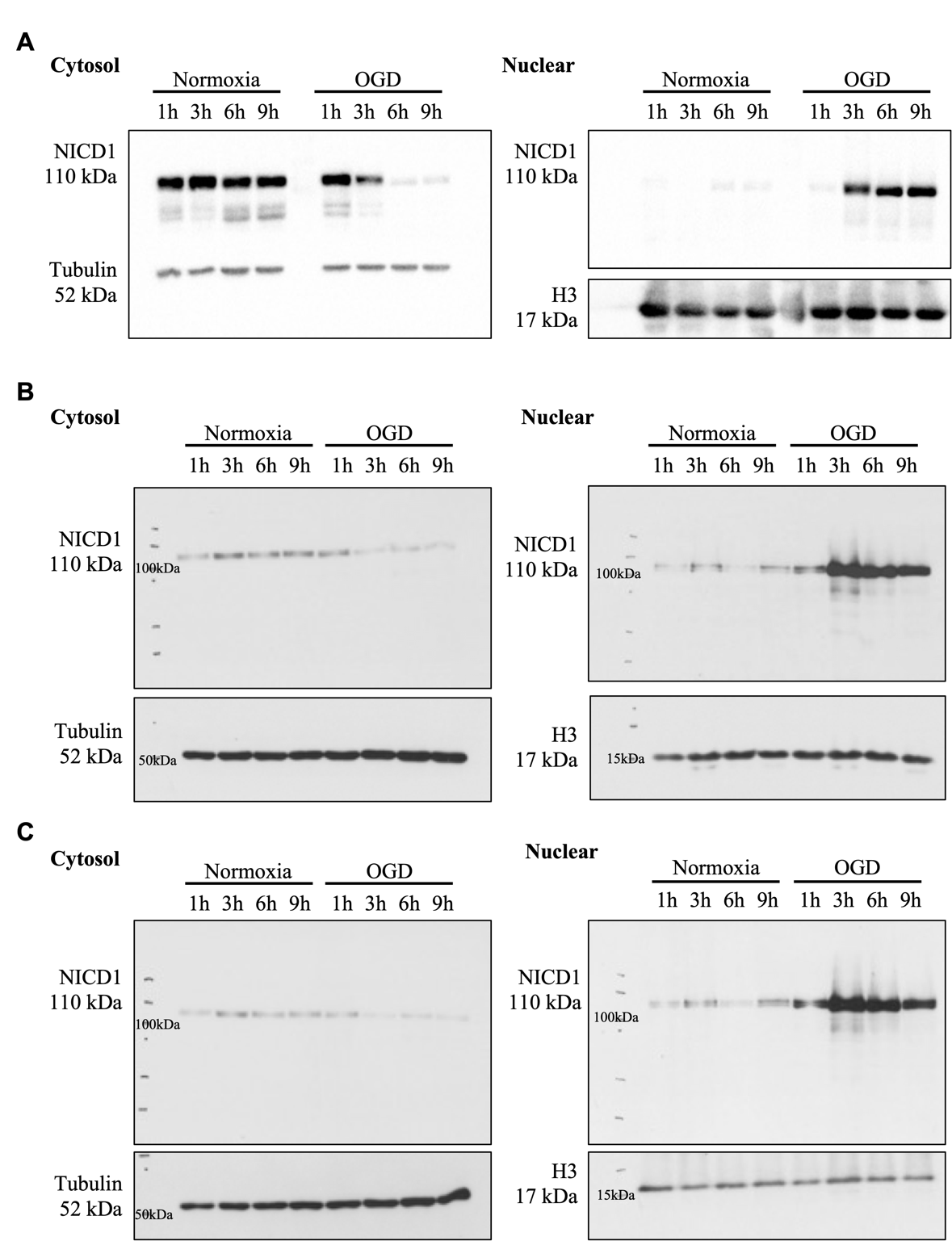


Figure S13. Immunoblots display NICD1 protein levels at different time points during OGD. (Fig. S1F) was based on three independent experiments, with (A), (B), and (C) representing each replicate.

**References**

1. Balaganapathy P, Baik SH, Mallilankaraman K, Sobey CG, Jo DG, Arumugam TV. Interplay between Notch and p53 promotes neuronal cell death in ischemic stroke. *J Cereb Blood Flow Metab*. Oct 2018;38(10):1781-1795. doi:10.1177/0271678x17715956

2. Kim HK, Cho J, Kim E, et al. Engineered small extracellular vesicles displaying ACE2 variants on the surface protect against SARS-CoV-2 infection. *J Extracell Vesicles*. Jan 2022;11(1):e12179. doi:10.1002/jev2.12179

3. Ng GY, Sheng D, Bae HG, et al. Integrative epigenomic and transcriptomic analyses reveal metabolic switching by intermittent fasting in brain. *Geroscience*. Aug 2022;44(4):2171-2194. doi:10.1007/s11357-022-00537-z

4. Dobin A, Davis CA, Schlesinger F, et al. STAR: ultrafast universal RNA-seq aligner. *Bioinformatics*. Jan 1 2013;29(1):15-21. doi:10.1093/bioinformatics/bts635

5. Li B, Dewey CN. RSEM: accurate transcript quantification from RNA-Seq data with or without a reference genome. *BMC Bioinformatics*. Aug 4 2011;12:323. doi:10.1186/1471-2105-12-323

6. Anders S, Huber W. Differential expression analysis for sequence count data. *Genome Biol*. 2010;11(10):R106. doi:10.1186/gb-2010-11-10-r106

7. Arumugam TV, Chan SL, Jo DG, et al. Gamma secretase-mediated Notch signaling worsens brain damage and functional outcome in ischemic stroke. *Nat Med*. Jun 2006;12(6):621-3. doi:10.1038/nm1403

8. Longa EZ, Weinstein PR, Carlson S, Cummins R. Reversible middle cerebral artery occlusion without craniectomy in rats. *Stroke*. Jan 1989;20(1):84-91. doi:10.1161/01.str.20.1.84

9. Ashwal S, Tone B, Tian HR, Cole DJ, Liwnicz BH, Pearce WJ. Core and penumbral nitric oxide synthase activity during cerebral ischemia and reperfusion in the rat pup. *Pediatr Res*. Oct 1999;46(4):390-400. doi:10.1203/00006450-199910000-00006
